# Mortality Communication and Expected Grief for Caregivers of Cancer Patients

**DOI:** 10.1192/j.eurpsy.2025.1787

**Published:** 2025-08-26

**Authors:** E. Orhan, C. H. Ayhan, F. Tanhan, M. C. Aktaş

**Affiliations:** 1 Department of Psychiatric Nursing, Van Yuzuncu Yil University Faculty of Health Sciences; 2 Guidance and Psychological Counseling, Van Yuzuncu Yil University , Van, Türkiye

## Abstract

**Introduction:**

Cancer is a disease with a high mortality rate and requires care. The identification of cancer with death may cause patients and caregivers not to talk about illness and death. However, communication is one of the most important elements of this disease process. In addition to the inability to talk, the increased responsibilities of caregivers cause physical, economic, social and psychological burdens on caregivers. This can lead to high levels of stress, mental fatigue and depression in caregivers. Poor communication between patients and caregivers is an important factor related to depressive symptoms in caregivers during the caregiving process. Open communication between caregivers and the patient is a factor that can positively affect the disease process. With this study, it can be found that being open communication can reduce depression, anxiety and stress levels in caregivers and support studies to reduce the burden of care of caregivers. Thus, it can be ensured that patients and their caregivers experience the disease process more positively and spend it more positively.

**Objectives:**

Existing measures of mortality communication may not capture much of the nuance in that cancer caregiver report to be particularly upsetting, so we thought it would be important to examine reliability and validity of the Caregiver’s Communication with the Patient about Illness and Death (CCID) Scale for measuring the extent to which caregivers of cancer patients discuss illness and death with the patientin Turkey.

**Methods:**

The methodological study will be conducted to establish the validity and reliability of the Caregiver’s Communication with the Patient about Illness and Death (CCID) Scaleto Turkish Culture and to determine the level of mortality communication, expected grief, care burden and mental health problems among cancer caregiver in Eastern Turkey. The sample of the study will consist of cancer caregivers and who agree to participate in the study. The data will be subjected to appropriate methods for statistical analysis and will be used to understand the relationships between mortality communication and grief, depression, anxiety, stress and caregiver burden at caregivers of cancer patients.

**Results:**

Data extraction is still on going in detailed style by principal authors. Description of studies and the key findings will be presented.

**Conclusions:**

It is predicted that not discussing illness and death causes an increase in depression, anxiety and stress levels in caregivers. In this study, it is aimed to evaluate depression, anxiety and stress in caregivers of cancer patients who will be evaluated with the scale and to evaluate their relationship with care burden.

**Disclosure of Interest:**

None Declared

